# Evaluation of the Accuracy of 99DOTS, a Novel Cellphone-based Strategy for Monitoring Adherence to Tuberculosis Medications: Comparison of DigitalAdherence Data With Urine Isoniazid Testing

**DOI:** 10.1093/cid/ciaa333

**Published:** 2020-03-28

**Authors:** Beena E Thomas, J Vignesh Kumar, M Chiranjeevi, Daksha Shah, Amit Khandewale, Kannan Thiruvengadam, Jessica E Haberer, Kenneth H Mayer, Ramnath Subbaraman

**Affiliations:** 1 Department of Social and Behavioural Research, National Institute for Research in Tuberculosis, Chennai, India; 2 Public Health Department, Municipal Corporation of Greater Mumbai, Mumbai, India; 3 Center for Global Health, Massachusetts General Hospital, Boston, Massachusetts, USA; 4 The Fenway Institute, Fenway Health, Boston, Massachusetts, USA; 5 Beth Israel Deaconess Medical Center, Boston, Massachusetts, USA; 6 Department of Public Health and Community Medicine and Center for Global Public Health, Tufts University School of Medicine, Boston, Massachusetts, USA; 7 Division of Geographic Medicine and Infectious Diseases, Tufts University School of Medicine, Boston, Massachusetts, USA

**Keywords:** tuberculosis, medication adherence, mHealth, digital adherence technologies, India

## Abstract

99DOTS is a cellphone-based strategy for monitoring tuberculosis medication adherence. In a sample of 597 Indian patients with tuberculosis, we compared 99DOTS’ adherence assessments against results of urine isoniazid tests collected during unannounced home visits. 99DOTS had suboptimal accuracy for measuring adherence, partly due to poor patient engagement with 99DOTS.

Poor tuberculosis (TB) treatment adherence is associated with increased risk of death, disease relapse, and development of drug resistance [1]. TB programs, including India’s, which has the largest epidemic globally, have historically monitored adherence using directly observed therapy (DOT), often using a facility-based approach. In this approach, patients regularly visit health facilities, where healthcare providers (HCPs) watch them take every dose [2]. However, DOT raises implementation and ethical challenges for health systems and patients and may not result in superior outcomes compared to self-administered therapy [2].

Digital adherence technologies (DATs) may facilitate patients taking medications at a place of their choice while having adherence monitored remotely [2, 3]. 99DOTS is a cellphone-based DAT that has been used to monitor > 150 000 TB patients in India’s government program since 2015, including most people living with human immunodeficiency virus (PLHIV) taking TB therapy in the public sector [4]. Medication blister packs are wrapped in a custom envelope. Dispensing each dose breaks a perforated flap in the envelope, revealing a phone number that the patient calls to report pill taking; a computer registers this event. HCPs visualize this real-time adherence record remotely to identify nonadherent patients [4].

While 99DOTS has advantages, its value for supporting treatment depends on its accuracy for measuring adherence. DATs may overreport adherence (ie, reporting pill ingestion when patients are actually nonadherent) or underreport adherence (ie, reporting pills being missed when patients are actually adherent) [2]. We present findings of a cohort study in India’s government TB program assessing 99DOTS’ accuracy by comparing its data against results of urine isoniazid tests collected during unannounced visits to patients’ homes.

## METHODS

Ethics committees at the National Institute for Research in TB, the Brigham and Women’s Hospital, and Tufts University approved this protocol.

To enroll a geographically diverse cohort, from August 2017 to February 2019, we sequentially enrolled and visited patients with drug-susceptible TB from 11 clinics in Mumbai (none of whom were PLHIV) and 5 clinics in Chennai and Vellore (all of whom were PLHIV). Patients were enrolled at different times in the treatment course, during treatment initiation or medication refill visits. We aimed to achieve representation of home visits across the first 2 months (intensive phase) and last 4 or more months (continuation phase) of therapy, since adherence and 99DOTS engagement may wane with clinical improvement.

At enrollment, we collected informed consent for a baseline questionnaire and future unannounced home visit. Patients became eligible for a home visit 3 weeks after enrollment to minimize short-term changes in 99DOTS engagement (ie, calling) or adherence from anticipation of the visit. The exact day of the visit was selected using a random number generator. During home visits, researchers administered a questionnaire and collected the patient’s urine sample for testing using the IsoScreen test (Supplementary Appendix).

Mixing urine with IsoScreen reagents results in a color change if isoniazid is in the sample; we classified such patients as “adherent per urine testing.” Samples without color change were classified as “nonadherent per urine testing.” Isoniazid is detectable in urine in nearly all patients 6–48 hours after ingestion and undetectable in nearly all patients > 72 hours after ingestion [5, 6]. However, urine isoniazid test results < 6 hours and 48–72 hours after ingestion are variable (the “gray zone”) [5, 6]. We excluded from analysis test results for patients whose 99DOTS record only reported doses taken within “gray zone” timings, without doses reported 6–48 hours before the home visit. This resulted in exclusion of 8% of test results.

Patients whose 99DOTS record reported at least 1 dose taken 6–48 hours before the home visit were classified as “adherent per 99DOTS.” Patients whose 99DOTS record reported that the last dose was taken > 72 hours before the visit were classified as “nonadherent per 99DOTS.” In addition to patient-reported doses (via phone calls), 99DOTS allows HCPs to report doses, which they do after calling patients to assess their adherence. We analyzed 99DOTS’ operating characteristics using only patient-reported doses and including both patient- and HCP-reported doses.

We estimated 99DOTS’ sensitivity, specificity, positive predictive value (PPV), negative predictive value (NPV), and accuracy for measuring adherence by comparing the 99DOTS record to urine isoniazid test results as the more rigorous biomarker-based comparator. We stratified findings by specific subgroups (eg, PLHIV vs non-PLHIV). We used the χ ^2^ test to assess differences in prevalence of medication adherence and 99DOTS operating characteristics between subgroups. We used McNemar test to assess differences in 99DOTS accuracy using patient-reported doses alone vs patient- and HCP-reported doses [7].

## RESULTS

Descriptive statistics and determination of the patient sample—including exclusion due to nonenrollment or unsuccessful home visit—are reported in the Supplementary Appendix (Table A and Figure A). We report results for 597 patients. Median age was 35 years (range, 18–83 years), and 346 (58%) were men; 203 (34%) were in the intensive phase, 287 (48%) were PLHIV, and 135 (23%) had a prior treatment history. Adherence (positive urine test) for the sample was 88% (95% confidence interval [CI], 85%–91%) (Table 1). Adherence and 99DOTS engagement were lower in PLHIV than non-PLHIV (both *P* ≤ .001). 99DOTS engagement was lower in the continuation phase than the intensive phase (*P* = .051); however, adherence across both phases was similar (*P* = .40).

**Table 1.** Prevalence of Tuberculosis Medication Adherence and Operating Characteristics of 99DOTS for Measuring Medication Adherence

Sample	No. of Patients in Sample	Prevalence of Adherence by Urine Isoniazid Testing^a^, % (95% CI)	Prevalence of Engagement With 99DOTS^b^, % (95% CI)	Sensitivity, % (95% CI)	Specificity, % (95% CI)	PPV, % (95% CI)	NPV, % (95% CI)	Accuracy (Proportion Correctly Classified), % (95% CI)
Overall cohort (patient-reported doses only)^c^	597	88 (85–91)	66 (62–70)	70 (66–74)	61 (48–72)	93 (91–95)	21 (18–25)	69 (65–72)
Overall cohort (patient- and provider-reported doses)	597	88 (85–91)	82 (78–85)	85 (81–88)	39 (28–52)	91 (90–93)	26 (20–33)	79 (76–82)
TB patients living with HIV^c^	287	83 (78–87)	60 (54–65)	65 (59–71)	66 (51–79)	90 (86–93)	28 (23–34)	65 (59–71)
TB patients without HIV infection^c^	310	93 (90–96)	72 (67–77)	74 (68–79)	48 (26–70)	95 (93–97)	12 (7–18)	72 (67–77)
TB patients in the intensive phase of therapy^c^	203	90 (85–94)	71 (65–77)	74 (68–80)	48 (41–55)	92 (89–96)	17 (12–22)	71 (65–77)
TB patients in the continuation phase of therapy^c^	394	87 (84–90)	64 (59–68)	68 (63–72)	66 (61–71)	93 (91–96)	23 (19–27)	68 (63–72)
TB patients without prior treatment history^c^	462	89 (86–92)	66 (61–70)	69 (64–73)	61 (46–75)	94 (91–96)	19 (15–23)	68 (64–72)
TB patients with a prior TB treatment history^c^	135	84 (76–90)	67 (59–75)	73 (63–81)	59 (36–79)	90 (84–94)	30 (21–40)	70 (62–78)

Abbreviations: CI, confidence interval; DOTS, directly observed therapy, short-course; HIV, human immunodeficiency virus; NPV, negative predictive value; PPV, positive predictive value; TB, tuberculosis.

^a^Refers to any dose of TB medications taken within 6–48 hours prior to the home visit, given the operating characteristics of the urine isoniazid test.

^b^Engagement with 99DOTS refers to any call made between 6 and 48 hours prior to the home visit.

^c^These operating characteristics were estimated only using patient-reported doses.

Using patient-reported doses, 99DOTS’ sensitivity, specificity, PPV, and NPV for measuring adherence were 70% (95% CI, 66%–74%), 61% (95% CI, 48%–72%), 93% (95% CI, 91%–95%), and 21% (95% CI, 18%–25%), respectively. Using patient- and HCP-reported doses, 99DOTS’ sensitivity increased (*P* ≤ .0001), but specificity decreased considerably (*P* ≤ .0001). In PLHIV, 99DOTS had lower sensitivity (*P* = .03) and PPV (*P* = .02) but higher NPV (*P* = .004) when compared to non-PLHIV.

## Discussion

In this study, medication adherence was relatively high, including among previously treated patients and in the continuation phase—subgroups for whom adherence is often assumed to be suboptimal. However, we likely overestimate daily adherence, given that the urine test is positive for most doses taken in the prior 48 hours. Also, posttreatment disease recurrence may increase even with mild nonadherence (≥ 10% of doses missed) [1]. Our findings therefore highlight the need to further improve adherence in this population, especially for PLHIV.

99DOTS engagement was consistently lower than adherence measured by the urine test—reflected in 99DOTS’ suboptimal sensitivity—particularly for PLHIV and in the continuation phase. As such, HCPs may face major challenges in using 99DOTS to identify nonadherence. 99DOTS’ low NPV suggests that HCPs have to reach out to about 10 patients reported as being nonadherent by 99DOTS to identify 2 patients who are actually not taking medications.

Levels of patient engagement with 99DOTS in our study are similar to findings from the broader 99DOTS rollout to > 20 000 patients in Mumbai [4]. Higher 99DOTS engagement has been reported elsewhere [8]; however, lack of an objective adherence measure and methodological differences make comparison with our study difficult. Prior studies suggest that suboptimal engagement with DATs is context dependent. For example, TB patients monitored using 2-way text messaging (SMS) in Pakistan [9] and individuals taking human immunodeficiency virus (HIV) preexposure prophylaxis monitored using electronic pillboxes in the United States [10]. both underreported adherence for different reasons, such as technology fatigue or concern about the pillbox’s conspicuous appearance, respectively.

99DOTS’ low specificity also indicates suboptimal benefits for identifying nonadherent patients. When using only patient-reported doses, 99DOTS missed identifying about 4 of 10 patients who were not taking medications, presumably because these patients called 99DOTS without ingesting doses. While other operating characteristics improved when HCP-reported doses were included, specificity decreased further, such that 99DOTS missed identifying 6 of 10 patients who were not taking medications. This finding may reflect patient hesitation to admit nonadherence when contacted by HCPs (ie, socially desirable responses).

Our study has important limitations. Because we achieved representation of home visits across the treatment course, our reported prevalence of adherence is roughly representative of adherence throughout therapy in the overall patient population. However, because we conducted a single home visit for each patient, our study was not designed to evaluate adherence throughout therapy for individual patients. Also, 22% of eligible patients did not enroll in our study or were not found despite multiple home visits. Enrolled participants may have been more motivated to engage with TB care. 99DOTS engagement and adherence may therefore be higher in our participants than in the broader patient population.

Forthcoming qualitative study findings will shed further light on patient engagement with 99DOTS. Future analyses should explore patient characteristics associated with adherence and 99DOTS engagement. Despite its suboptimal accuracy, 99DOTS may still improve adherence or treatment outcomes via other pathways, such as facilitating habit formation in pill taking. Rigorous trials are needed to assess 99DOTS’ impact on these outcomes.

In summary, we found suboptimal accuracy of 99DOTS for measuring TB medication adherence in a multisite cohort study. Our study highlights benefits of urine isoniazid testing for understanding adherence and assessing the accuracy of DATs, especially given growing interest in these technologies [2, 3]. Our findings raise concerns about India’s large-scale 99DOTS deployment and highlight urgent need for high-quality studies regarding its impact on treatment outcomes, especially given that a recent study found that HIV clinics implementing 99DOTS had higher unsuccessful TB treatment outcomes than clinics that had not [11]. Our findings highlight a need to strengthen 99DOTS’ implementation, with ongoing evaluation of whether its accuracy—and value for monitoring adherence—can be improved, or whether alternative strategies such as testing for urine isoniazid or other biomarkers should be studied for routine monitoring of medication adherence.

## Supplementary Data

Supplementary materials are available at *Clinical Infectious Diseases* online. Consisting of data provided by the authors to benefit the reader, the posted materials are not copyedited and are the sole responsibility of the authors, so questions or comments should be addressed to the corresponding author.

ciaa333_suppl_Supplementary_Appendix

## Notes

***Acknowledgments.*** The authors thank all of the participants who took part in this study. We acknowledge the immense contribution of all the field investigators for their efforts in collecting data. The Mumbai team included Gunjan Rahul Gaurkhede, Apurva Shashikant Walgude, Yogesh Nivrutti Salve, Jagannath Dattatraja Kumbar, Kamble Rakesh Suresh, Shweta Shantaram Bagade, and Meena Atul Kamble. The Tamil Nadu team included J. Hephzibah Mercy, C. Jeganathan, S. Yokeshwaran, S. Kokila, K. Sathiyamoorthy, E. Michael Raj, P. Brindadevi, K. Jegan, B. Sathyan Raj Kumar, Mariyamma Paul, and Ranjith Kumar. The authors also appreciate statistical advice provided by Professor Misha Eliasziw, Tufts University School of Medicine.

***Disclaimer.*** The funding bodies had no role in study design, data collection, data analysis, data interpretation, or manuscript writing.

***Financial support.*** This research was primarily supported by grants from the Bill & Melinda Gates Foundation (grant numbers OPP1154670 and OPP1154665). R. S. also received support from a Harvard Catalyst KL2/Catalyst Medical Investigator Training Award (grant number KL2 TR001100); the Harvard Center for AIDS Research (grant numbers 5P30AI060354-13); and a Doris Duke Clinical Scientist Development Award (grant number 2018095). J. E. H. received support from the National Institutes of Health during the conduct of the study (grant number K24MH114732).

***Potential conflicts of interest.*** B. E. T., J. V. K., C. M., A. K., and R. S. are conducting research evaluating the implementation of 99DOTS and evriMED (a digital pillbox) in India, supported by the Bill & Melinda Gates Foundation; none of them have any financial interest in these technologies. J. E. H. is currently conducting research on the Wisepill device and evriMED1000 (digital pillboxes); she has no financial interest in these technologies. J. E. H. also reports personal fees from Merck, outside the submitted work. All other authors report no potential conflicts of interest. All authors have submitted the ICMJE Form for Disclosure of Potential Conflicts of Interest. Conflicts that the editors consider relevant to the content of the manuscript have been disclosed.
