# Supplementary material for: Evaluation of the Accuracy of 99DOTS, a Novel Cellphone-based Strategy for Monitoring Adherence to Tuberculosis Medications: Comparison of DigitalAdherence Data With Urine Isoniazid Testing
Source: Clin Infect Dis. 2020 Mar 28;71(9):e513–6. doi: 10.1093/cid/ciaa333 (PMC7713673; doi:10.1093/cid/ciaa333)
Supplement: ciaa333_suppl_Supplementary_Appendix [file ciaa333_suppl_supplementary_appendix.docx]

**Supplementary Appendix**

**Supplement to:**

*Evaluation of the accuracy of 99DOTS, a novel cellphone-based strategy for monitoring adherence to tuberculosis medications: Comparison of digital adherence data with urine isoniazid testing*

Beena E. Thomas, J. Vignesh Kumar, Chiranjeevi M., Daksha Shah, Amit Khandewale, Kannan Thiruvengadam, Jessica E. Haberer, Kenneth H. Mayer, Ramnath Subbaraman

**Correspondence:** B.E. Thomas, Department of Social and Behavioural Research, ICMR- National Institute for Research in Tuberculosis, No. 1, Mayor Sathiyamoorthy Road, Chetpet, Chennai – 600 031, India ([beenaelli09@gmail.com](mailto:beenaelli09@gmail.com)). Phone: +91-44-2836-9525

**Methods**

**Types of information collected in study questionnaires**

The baseline questionnaire collected at the time of patient enrollment into the study assessed patient demographics, cellphone accessibility, and psychosocial data, including a basic depression screen (Patient Health Questionnaire-2) and basic alcohol use screen (AUDIT-C). The questionnaire collected at the time of the home visit with the patient assessed self-reported medication adherence using four-day recall, reasons for non-engagement with 99DOTS, and reasons for medication non-adherence.

**Reason for using urine isoniazid testing as a measure of adherence**

Studies have used different biomarkers for assessing medication adherence. For drug-susceptible tuberculosis (TB) patients, the most common biomarker-based measures have focused on testing for isoniazid or rifampin content in urine or serum. We assessed various urine-based testing options, given challenges around collecting blood samples from patients.

Our literature review suggested considerable challenges in using rifampin as a biomarker for measuring adherence. First, pharmacokinetic studies suggest that rifampin is cleared from plasma (and therefore not present in urine) within <12 hours in many patients [1]. As such, depending on the timing of the unannounce home visit in relation to pill consumption, urine rifampin has the potential to indicate a patient is non-adherent even when the patient is actually correctly taking medications. Multiple prior studies affirm that the pharmacokinetic profile of rifampin leads measurement of this biomarker to misclassify adherent patients as being non-adherent. For example, in one study, only 20% of TB patients who were truly adherent were correctly identified as being adherent when the standard orange-reddish discoloration that can happen with rifampin ingestion was used as a measure [2]. Other studies that use more sophisticated biochemical tests to assess for rifampin content in urine found only 43% to 64% sensitivity for correctly identifying true medication adherence [3,4].

In contrast to the poor sensitivity of urine rifampin for measuring adherence to TB medications, urine isoniazid testing has high sensitivity for observed TB medication adherence for doses taken within the prior 48 hours [3-6]. The test also has high specificity for non-adherence for patients who have not taken any doses in the prior 72 hours [3-6]. As described in the main manuscript, we assumed that doses taken within 6 hours of urine isoniazid testing may have variable test results, because the dose may take time to get absorbed by the gastrointesintal tract. Notably, urine isoniazid test characteristics are not significantly affected by whether a patient is a slow or fast acetylator of isoniazid [6]. Given the availability of a point-of-care test for assessing urine for isoniazid content (IsoScreen, GFC Diagnostics, UK), we found urine isoniazid testing to be a rigorous comparator against which the accuracy of 99DOTS for measuring adherence to TB medications could be assessed.

**Results**

**Determination of final patient sample for the accuracy analysis**

Out of 832 tuberculosis (TB) patients at all sites who met eligibility criteria for this study, 84 (10%) patients either did not consent for the study (53 patients) or were not physically available to enroll because a family member was picking up the TB medication refill on their behalf (31 patients). Of the 748 who enrolled in the study and completed the baseline questionnaire, we were not able to complete an unannounced home visit despite at least three attempts for 98 (13%). Of the remaining 650 patients for whom home visits were completed, 53 (8%) were excluded from analysis because their 99DOTS call patterns fell within the “gray zone” where urine isoniazid test results could be variable (Figure A).

**
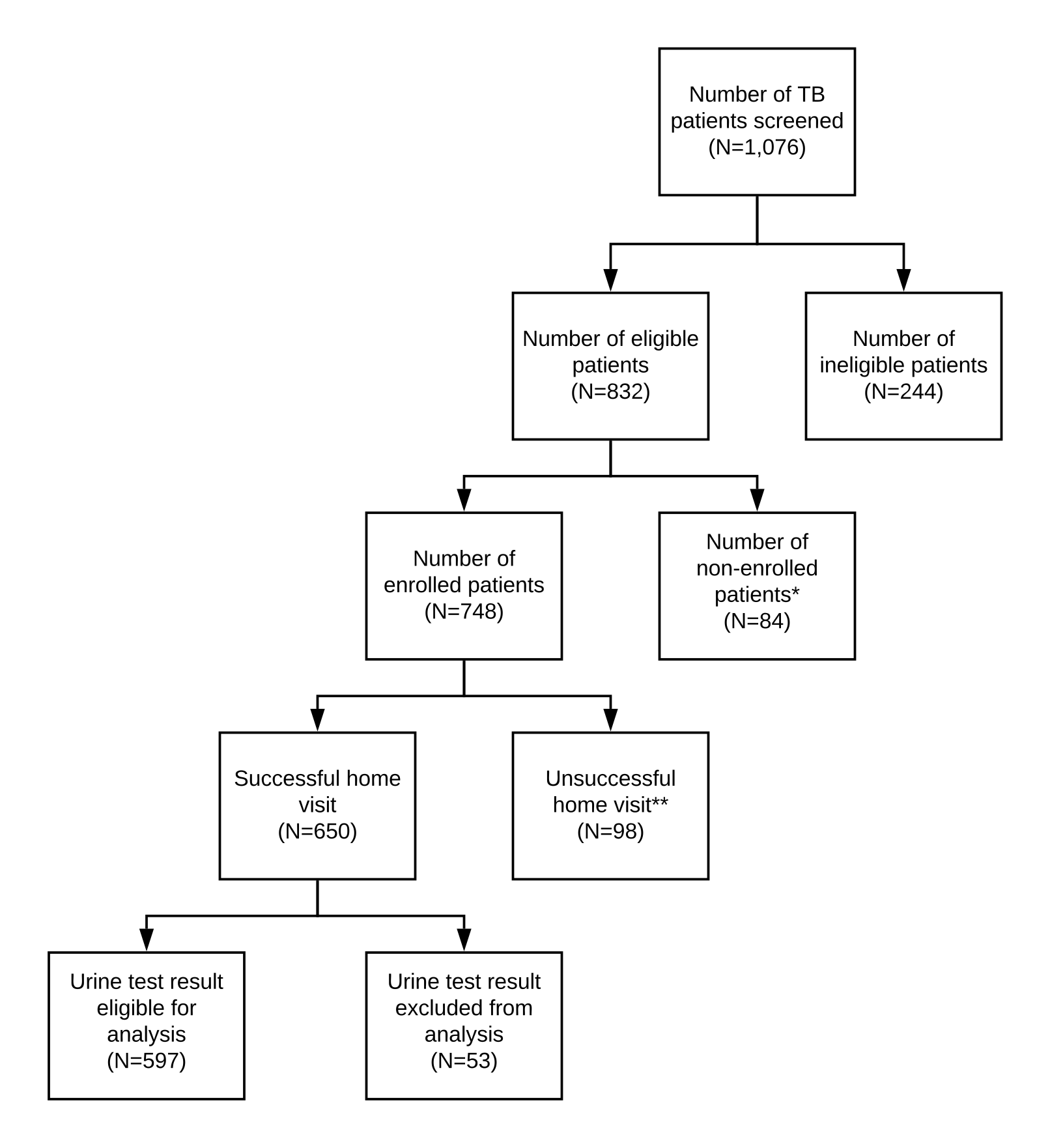
**

**Figure A. Determination of final patient sample for accuracy analysis**

*Reasons for non-enrollment included not consenting for the study and the patient not being available because a family member was picking up their medication refill

**We only considered home visit attempts to be “unsuccessful” after three attempts were made to complete the home visit

**Descriptive characteristics of the final patient sample**

We describe the descriptive characteristics for the final patient sample below. Key aspects of these findings are described in the results section of the main manuscript. Home visits were conducted throughout all six or more months of TB treatment; however, a smaller proportion were conducted in the first month of therapy, because we waited at least three weeks prior to conducting a home visit. The majority of treatment outcomes (71.2%) were favorable (i.e., cure or treatment completion) and the remaining outcomes were unfavorable or not reported.

**Table A. Descriptive characteristics of the final patient sample (n=597)**

| **Characteristic** | **TB patients living with HIV (n=287)**  **N (%)** | **TB patients without HIV infection (n=310)**  **N (%)** | **Overall cohort (n=597)**  **N (%)** |
| --- | --- | --- | --- |
| **Sex** |  |  |  |
| Male | 195 (67.9) | 151 (48.7) | 346 (58.0) |
| Female | 92 (32.1) | 159 (51.3) | 251 (42.0) |
| **Age** |  |  |  |
| 18 - 25 years | 16 (5.6) | 141 (45.5) | 157 (26.3) |
| 26 - 35 years | 51 (17.8) | 91 (29.4) | 142 (23.8) |
| 36 - 45 years | 120 (41.8) | 38 (12.3) | 158 (26.5) |
| 46 - 55 years | 70 (24.4) | 23 (7.4) | 93 (15.6) |
| 56+ years | 30 (10.5) | 17 (5.5) | 47 (7.9) |
| **Prior treatment history** |  |  |  |
| Prior treatment history (Category 1) | 228 (79.4) | 234 (75.5) | 462 (77.4) |
| Prior treatment history (Category 2) | 59 (20.6) | 76 (24.5) | 135 (22.6) |
| **Treatment phase of home visit** |  |  |  |
| Intensive phase | 108 (37.6) | 95 (30.6) | 203 (34.0) |
| Continuation phase | 179 (62.4) | 215 (69.4) | 394 (66.0) |
| **Treatment month of home visit** |  |  |  |
| Month 1 | 46 (16) | 10 (3.2) | 56 (9.4) |
| Month 2 | 55 (19.2) | 72 (23.2) | 127 (21.3) |
| Month 3 | 61 (21.3) | 65 (21) | 126 (21.1) |
| Month 4 | 40 (13.9) | 68 (21.9) | 108 (18.1) |
| Month 5 | 36 (12.5) | 44 (14.2) | 80 (13.4) |
| Month 6 | 22 (7.7) | 25 (8.1) | 47 (7.9) |
| Beyond month 6^a^ | 27 (9.4) | 26 (8.4) | 53 (8.8) |
| **Treatment outcomes** |  |  |  |
| Cured | 81 (28.2) | 74 (23.9) | 155 (26.0) |
| Treatment completed | 111 (38.7) | 205 (66.1) | 316 (52.9) |
| Treatment failure or regimen change | 5 (1.7) | 6 (1.9) | 11 (1.8) |
| Lost to follow up | 11 (3.8) | 13 (4.2) | 24 (4.0) |
| Died | 15 (5.2) | 1 (0.3) | 16 (2.7) |
| Not evaluated / not reported^b^ | 64 (22.3) | 11 (3.6) | 75 (12.6) |

^a^Because many patients in India’s TB program undergo extension of TB treatment beyond 6 months, some patients enrolled late in the continuation phase of therapy had home visits conducted beyond month 6 of therapy.

^b^Some patients in our cohort did not have treatment outcomes reported in Nikshay, the government’s official TB database as of February 2020; this proportion of patients without reported outcomes is similar for the larger sample of patients taking treatment in the public sector during 2017-2018 at our study sites and these outcomes could be reported later from paper records.

**References**

1. Ruslami R, Nijland HM, Alisjahbana B, Parwati I, van Crevel R, Aarnoutse RE. Pharmacokinetics and tolerability of a higher rifampin dose versus the standard dose in pulmonary tuberculosis patients. Antimicrob Agents Chemother. 2007;51(7):2546-51. doi: 10.1128/aac.01550-06. PMID: 17452486.

2. van den Boogaard J, Lyimo RA, Boeree MJ, Kibiki GS, Aarnoutse RE. Electronic monitoring of treatment adherence and validation of alternative adherence measures in tuberculosis patients: a pilot study. Bull World Health Organ. 2011;89(9):632-9. doi: 10.2471/blt.11.086462. PMID: 21897483.

3. Meissner PE, Musoke P, Okwera A, Bunn JE, Coulter JB. The value of urine testing for verifying adherence to anti-tuberculosis chemotherapy in children and adults in Uganda. Int J Tuberc Lung Dis. 2002;6(10):903-8. PMID: 12365577.

4. Whitfield R, Cope GF. Point-of-care test to monitor adherence to anti-tuberculous treatment. Ann Clin Biochem. 2004;41(Pt 5):411-3. doi: 10.1258/0004563041731637. PMID: 15333195.

5. Elizaga J, Friedland JS. Monitoring compliance with antituberculous treatment by detection of isoniazid in urine. Lancet. 1997;350(9086):1225-6. doi: 10.1016/s0140-6736(05)63457-5. PMID: 9652571.

6. Soobratty MR, Whitfield R, Subramaniam K, Grove G, Carver A, O'Donovan GV, et al. Point-of-care urine test for assessing adherence to isoniazid treatment for tuberculosis. Eur Respir J. 2014;43(5):1519-22. doi: 10.1183/09031936.00132613. PMID: 24435008.
